# Co-Application of Sheep Manure and Azotobacter Biofertilizer Enhances Growth, Yield, Essential Oil Profile, and Antioxidant Activity in Summer Savory

**DOI:** 10.3390/biology14081096

**Published:** 2025-08-21

**Authors:** Ugur Benli, Gulsum Yaldiz, Mahmut Camlica

**Affiliations:** Agriculture Faculty, Field Crops Department, Bolu Abant İzzet Baysal University, 14280 Bolu, Türkiye; ugurben98@gmail.com (U.B.); mcamlica25@gmail.com (M.C.)

**Keywords:** organic manure, medicinal plant, savory, azotobacter, chemical property, plant production

## Abstract

Overuse of chemical fertilizers can threaten the agro-ecological balance, including an excessive accumulation of certain elements, such as nitrogen and phosphorus. On the other hand, organic fertilizers and biofertilizers, which are eco-friendly and cost-effective, increase biological nitrogen fixation and enhance the availability of nutrients to plants. The aim of this research was to study the possibility of using a full (22.50 t/ha) and 50% (11.25 t/ha) treatment of sheep manure with azotobacter (100 mL/20 L) instead of inorganic fertilizers for increasing savory (*Satureja hortensis* L.) growth production and yield value as well as improving chemical and biological properties. The results showed that the treatment with 50% sheep manure recorded the highest total dry herb (3.18 t/ha) yield. The inorganic fertilizer resulted in the highest essential oil content (1.43% *v*/*w*) and γ-terpinene (10.38% *v*/*v*), cymol (5.90% *v*/*v*), and α-bisabolene (5.28% *v*/*v*) values. The maximum carvacrol value (42.54% *v*/*v*) was recorded in the savory herb after applying no fertilization to the plants, while the highest concentration of thymol (16.09% *v*/*v*) was obtained by applying the full sheep manure treatment. The full sheep manure + azotobacter treatment had the highest mean α-terpinene value (7.22% *v*/*v*), and the 50% sheep manure + azotobacter treatment had the highest mean α-phellandrene value (6.44% *v*/*v*). The highest DPPH activity (60.86%) and FRAP value (69.64 mg TE/g DW) were observed with the azotobacter + full sheep manure treatment, while the highest total phenolic content (96.87 mg GAE/g DW) and total flavonoid content (45.97 mg QE/g DW) in the savory herb were obtained from the combination treatment of 50% sheep manure doses + azotobacter. Principal coordinate analysis (PCA) revealed distinct clustering of treatments, with PC1 and PC2 explaining >60% of the variance, highlighting the dominant role of sheep manure doses in morphological/yield properties. Heatmap analysis grouped the treatments (right) and examined properties (bottom) as two main groups. The full sheep manure + biofertilizer and inorganic fertilizer treatments were found in the first group, depending on the treatments. Moreover, the heatmap analysis revealed that the full and 50% sheep manure (SM) treatments played critical roles in separating the examined properties, and the DPPH and carvacrol properties were grouped together compared to other properties. Thus, the results suggest that treatment with azotobacter could be employed in combination with appropriate rates of sheep manure to obtain the maximum benefits regarding herb yield, biological activity, and essential oil components.

## Simple Summary

Organic manures can increase soil properties such as pH, maintain soil health, and enable landowners to farm with organic or ecological status instead of chemical fertilizers. So, organic manures have gained an important place as an alternative to chemical fertilizers in agricultural areas. In this context, sheep manure and biofertilizer treatments can be used to improve soil properties and plant production. In this study, different doses of sheep manure were applied to savory plants with a biofertilizer (azotobacter) treatment. The results showed that both sheep manure doses and azotobacter treatments increased the yield and quality properties of savory. In particular, half doses of sheep manure increased the fresh and dry herb weight values, while the full sheep manure doses improved the essential oil compositions. The mixture of the sheep manure doses and azotobacter had positive impacts on the bioactive compounds of the savory. In conclusion, it is reported that these treatments can be used for savory production in sustainable agriculture.

## 1. Introduction

*Satureja hortensis* L. (savory) is an aromatic plant from the Lamiaceae family, and its essential oils are used in various treatment areas, from the food industry to aromatherapy [1]. Polyphenols and flavonoids, which are responsible for the antioxidant, antimicrobial, antiparasitic, pesticidal, anti-inflammatory, analgesic, hepatoprotective and anticancer properties, dominate the compounds in natural products obtained from savory extracts and essential oils [2]. Many studies have shown that savory herb is a good natural antioxidant source due to its high phenolic content and antioxidant activity [3]. In Türkiye, dried savory herb is used as an herbal tea and condiment in addition to being utilized in folk medicine to treat asthma, colic, bronchitis, and coughs, and it is used in the food industry as a flavoring, aromatic, and preservative agent [3].

The essential oils of savory contain high amounts of phenolic compounds, such as carvacrol, γ-terpinene, thymol, p-cymen, β-caryophyllene, linalool, and other terpenoids. However, some reports have been published based on the differences in the composition of essential oils of *S. hortensis* species [4,5,6]. In addition, it has been reported that the essential oils of savory species that have a high carvacrol and thymol content have a stronger antimicrobial effect and are more suitable for use in the pharmaceutical industry [4].

Excessive and long-term treatment of chemical fertilizers has resulted in various environmental issues, such as soil degradation, water eutrophication, and nitrogenous gas emissions [7]. Furthermore, crop yields have decreased significantly in recent years owing to the rapid decline in soil quality. Therefore, improving soil quality and boosting crop yields are urgent issues that need to be addressed, and organic fertilizers are a viable solution. Using an optimum dose of organic fertilizer instead of chemical fertilizers can improve soil pH, sustain soil health, and enable landowners to practice organic or ecological farming [8].

In general, organic and bio-organic fertilizers affect the structure and diversity of bacterial and fungal communities in their soils, promoting the release and transformation of soil nutrients, which is beneficial for the nutrient supply and the soil quality improvement. Soil microorganisms represent an important component in evaluating soil quality, serving as a biological indicator or as a sustainability index for production systems. [8,9]. Therefore, emphasis is now placed on using biofertilizers in crop production, such as biological nitrogen fixers (azotobacter/Azospirillum/Rhizobium) [9]. These biofertilizers improve the plant growth by supplying nutrients, producing vitamins (e.g., thiamine and riboflavin) and plant hormones (namely indole acetic acid (IAA) and gibberellins (GA)), which could enhance nutrient absorption and photosynthesis [10,11].

It has been reported that incorporating organic fertilizers, such as bio-organic fertilizers and sheep manure, into plant cultivation will reduce the overuse of chemical fertilizers and reduce environmental pollution, as well as producing high-quality agricultural products free of harmful agrochemicals, which will ensure safety for human consumption [12,13]. Additionally, organic fertilizer treatments are vital for maintaining ecosystem stability, as they enhance plant growth, nutrient uptake, soil microbial diversity, and yield [14,15]. Although previous studies have investigated the effects of sheep manure on morphological traits, yield, and quality parameters in savory [16], as well as the effects of biofertilizers and chitosan treatments [17], the combined effects of sheep manure and biofertilizer on savory have not been studied. Therefore, in order to maximize the potential of organic savory production systems, it is important to improve soil nutrient effectiveness as well as soil microbial abundance and activity [18,19].

In this study, savory was cultivated with the full as well as half recommended doses of sheep manure alone and in combination, with biofertilizer (azotobacter) inoculation, with biofertilizer inoculation alone, the recommended dose of inorganic fertilizer and unfertilized treatments.

Based on the above findings, we hypothesized that optimizing sheep manure doses and azotobacter may decrease the demand for inorganic fertilizer use in savory production and co-composting by combining reduced sheep manure doses with azotobacter inoculation. This approach may fulfill savory’s P and N requirements and, consequently, improve soil health through organic matter addition. Therefore, this study aimed (I) to reduce reliance on inorganic fertilizers in savory cultivation, mitigating environment impacts while improving savory growth, and (II) to identify the performance effects of azotobacter inoculation combined with full or half sheep manure doses on savory yield and quality.

## 2. Materials and Methods

### 2.1. Plant Material and Treatments

In this study, *Satureja hortensis* L. seeds were obtained from Yalova Seed Company, (Yalova, Türkiye), and they were sown in pots (400 mm diameter) in the climate room (27 °C—65% humidity) located in the Field Crops Department of the Faculty of Agriculture of Bolu Abant İzzet Baysal University in April 2023, and savory seedlings were obtained. The obtained seedlings (5–6 leaves, about 10 cm) were planted in the research and application area (40°44′44″ N, 31°37′45″ E, 881 m above sea level) of the Field Crops Department of the Faculty of Agriculture of Bolu Abant İzzet Baysal University in May 2023. Before the seedlings were planted, full-dose sheep manure doses (22.50 t/ha) and 50% dose sheep manure (11.25 t/ha) were applied to the plots at a depth of 10–15 cm and mixed with the soil using disc harrow.

Seven treatments from the factorial combination of the described sheep manure rates and biofertilizer treatments were arranged in a randomized complete block design (RCBD) with three replicates, resulting in 21 experimental plots (Table 1).

In this study, there were seven treatments, described as follows.

With planting seedlings, ammonium sulphate (60 kg/ha) was split-applied, 50% at transplanting, 50% after first cut, and diammonium phosphate (DAP) fertilizer was applied at a rate of 40 kg/ha at transplanting, providing 18% nitrogen (N) and 46% phosphorus (P_2_O_5_) as an inorganic fertilizer. Unfertilized plants were not treated with any biofertilizer and sheep manure. In studies on SM treatment, to ensure high quality and yield, this manure was recommended to be used at doses of 22.50 t/ha in *Foeniculum vulgare* L. [20] and 20 t/ha in *Triticum aestivum* L. [21]. Similarly, effective nitrogen dose was reported as 0.06 t/ha for basil [22] and dill [23].

The chemical properties of the sheep manure are shown in Table 2.

**Figure 1:**
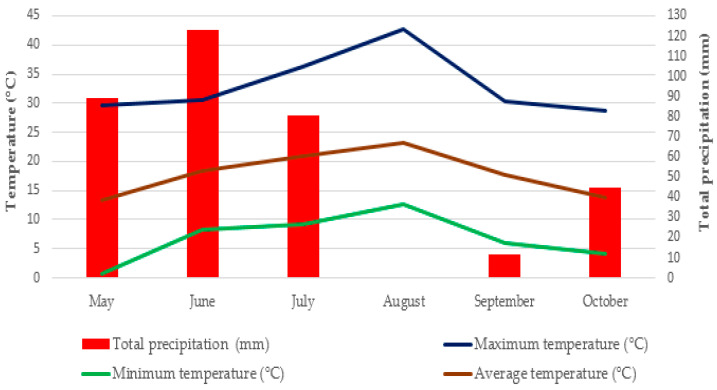
Climatic data of the experiment during plant growth period.

Each experimental plot consisted of five rows, with a distance of 0.3 m between each row and 0.2 m between each plant. In addition, the plot size was 4.5 m, spacing between plots was 1 m, and spacing between blocks was 2 m. All necessary drip irrigation and maintenance practices were applied from the transplanting of seedlings through to the last cutting. The irrigation times were slightly adjusted according to the weather, and plants were watered every 2–3 days using a drip irrigation system. When temperatures exceeded 28 °C, daily irrigation was applied (Figure 1). Plants were hand-harvested at 50% flowering onset. Cuttings were made at nearly 10 cm above the ground for fast regrowth, and cutting of savory was performed in warm and sunny weather to allow for high essential oil yield. Savory was cut three times between 17 July and 8 August for first cut, 23 August and 8 September for second cut, and 27 September and 4 October at the 50% flowering days.

The soil properties of the experimental area were as follows: medium in phosphorus (75.80 kg/ha), rich in potassium (947.4 kg/ha) and low in organic matter (1.06%), clayey (67.32%) and neutral pH (7.46), medium lime (7.37%), and low salinity (0.04%) [24]. During the vegetation period (May–October), the experimental area had a mean temperature of 17.88 °C and total precipitation of 349.4 mm [25]. Detailed climatic information is given in Figure 1.

#### 2.1.1. Biofertilizer and Inoculum Preparation

The commercial Vitormone plus drip of Bioglobal Anonim company (Antalya, Türkiye) was used in azotobacter treatments. The mixture was composed of *Azotobacter chroococcum* and *Azotobacter vinelandii* (10^7^ CFU/g). The pH scale was in the range of 6 to 8. Recommended fertilizer amounts were as follows: the mixture of 100 mL Vitormine plus drip was added to 20 L water and homogenized. The roots of seedlings were kept in this mixture for 30 min before planting [26].

#### 2.1.2. Measurement and Determinations

The different parameters studied were 50% flowering days, plant height (cm), number of branches (number/plant), yield (t/ha), essential oil components, antioxidant activities. The 50% flowering days were determined from seedlings to first cut days for first cut, from first cut to second cut for second cut and from second cut to third cut for third cut. After each cut, the cutting plants were weighed and calculated as fresh herb weights in t/ha. Then, these fresh herbs were dried at the prescribed drying temperature (35 °C) in thermal drying compartment.

### 2.2. Essential Oil Isolation and Gas Chromatography/Mass Spectrometry Analysis

Essential oil contents were determined volumetrically with the Clevenger apparatus according to the water distillation method in dried herbs at 35 °C. Approximately 20 g of samples from the dried herbs prepared for analysis was weighed. The weighed sample was placed in a glass flask (500 mL). Approximately 10-times the amount of sample (200 mL) of pure water was added. It was subjected to hydro distillation for approximately 4 h. Then, the reading of the essential oil sample that accumulated in the graduated section and created a phase difference with water was made and the result was recorded in mL. Then, the essential oil content was calculated as a percentage based on the weighed amount as mL/100 (% *v*/*w*) g [27].

Essential oil component analysis of samples was carried out using a GC/GC-MS (gas chromatography (Thermo Scintific Trace 1300)–mass detector (Thermo Scientific ISQ QD, Waltham, MA, USA)) device and a capillary column (TG-624; 30.0 m × 0.25 mm × 1.4 μm). Samples were diluted 1:100 with hexane for analysis. Helium was used as the carrier gas at a flow rate of 1.00 mL/min in the analysis, and samples were injected into the device at 1 μL. The injector temperature was kept at 230 °C, the column temperature program was set as 40 °C (2 min), from 40 °C to 220 °C at 8 °C/min and 220 °C (10 min) and in splitless mode. The total analysis time was 36 min, in line with this temperature program. For the mass detector, the scanning range (*m*/*z*) was 40–450 atomic mass units, the electron bombardment ionization was 70 eV, the transfer line temperature was 280 °C and the ion source temperature was 220 °C, and the data from WILEY and NIST libraries were used to identify the components of the essential oil. The results were made using MS detectors for the percentages of the components and the identification of the components [27].

### 2.3. Polyphenol Extraction and Analyses

#### 2.3.1. Extract Preparation

Herb extraction from savory was performed following the method described by Gikas et al. [28], with slight modifications. Briefly, 10 g of plant herbs was ground and extracted using shaking water bath (Miprolab, Ankara, Türkiye) with 80% methanol at 30 °C for 60 min. The mixture was then filtered, and the residue was reconstituted with 80% methanol to a final volume of 100 mL. Extracts were stored at +4 °C until further analysis. The extracts were used for the total phenolic and flavonoid contents with antioxidant activities.

#### 2.3.2. Total Phenolics

Total phenolic contents of the savory extracts were determined according to the method reported by Singleton et al. [29]. Thus, 0.4 mL of distilled water and 0.5 mL of diluted Folin-Ciocalteu reagent (10% *v*/*v*) were added to 100 μL of savory extract. These extracts were left to stand for 5 min, and then 1 mL of 7.5% sodium carbonate (*w*/*v*) was added. Absorbances were measured at 765 nm wavelength using a spectrophotometer (Drawell Instrument Co., Ltd., Chongqing, China) after 2 h. The calibration curve of gallic acid (GA) was used to estimate the sample activity capacity. The results were recorded as mg GA equivalent per g of dry sample (mg GA/g DW).

#### 2.3.3. Total Flavonoids

Total flavonoid contents were determined according to Chang et al. [30] and were determined by taking into account the protocol reported. The 1 mL extract, 4 mL distilled water and 300 µL NaNO_2_ (0.3%) mixture was shaken for five minutes. Then, 300 µL AlCl_3_ (10%) and 200 µL 1 M NaOH were added to this mixture and mixed. In the last stage, 2.4 mL distilled water was added and shaken well. The absorbance of total flavonoid content was determined at 510 nm. Quercetin compound was used as a standard in determining the total flavonoid content, and the results were reported as mg QE/g DW.

#### 2.3.4. Antioxidant and Reducing Activity

The radical scavenging activity of savory extract was determined using 1,1-diphenyl-2-picrylhydrazyl (DPPH) assay [31] with some modifications. Thus, 1 mL of extract sample was mixed with 2 mL of DPPH radical solution (1 mg DPPH in 100 mL methanol). After mixing well and incubating at room temperature for 5 min, absorbance was measured at 517 nm. As a control, 2 mL of DPPH solution was dissolved in 1 mL of distilled water. Free radical scavenging activity (RSA) was calculated using the following equation.

RSA (%) = [(A_control − A_sample)/A_control] × 100

FRAP method was determined by the method suggested by Benzie and Strain [32]. First, 300 mM acetate buffer FRAP reagent pH: 3.6 (3.1 g sodium acetate trihydrate + 16 mL glacial acid 1:1 distilled water); 10 mM 2,4,6-tris (2-pyridyl)-striazine (TPTZ) in 40 mM HCl; and 20 mM FeCl_3_ 6H_2_O 10:1:1 ratio was re-prepared to provide the working reagent. Additionally, 1 mL of FRAP active was added to 100 μL of savory extract, and absorbances were determined in the spectrophotometer after 30 min at 595 nm wavelength. Trolox calibration curve (TE) was created to approximately determine the activity capacity of the sample, and the results were recorded as mg of trolox per g of dry sample (mg TE/g DW).

### 2.4. Statistical Analysis

Statistical analysis was determined by analysis of variance in accordance with the randomized complete block design. Statistical analysis was performed in XLSTAT Microsoft Excel 2016 program (https://www.xlstat.com) to determine the differences between the means of the investigated traits using the Least Significant Difference (LSD) test at the level of *p* < 0.05. The F-statistics, degrees of freedom, and *p*-values are given in the Supplementary Tables (Table S1). Principal coordinate and heatmap analysis was performed to determine the relationships between the examined properties of treatments using the JMP 14 software and ClustVis 2.0. programs, respectively.

## 3. Results and Discussion

### 3.1. Morphological Parameters

The following morphological parameters were measured: the 50% flowering days, plant height and number of branches. As can be seen from Table 3, the 50% flowering day of different fertilizer treatments was found to be statistically significant at the level of 0.05% (*p* < 0.05). Among different treatments, the mean 50% flowering day varied between 32.33 and 36.39 days. Considering the mean cuts, the latest 50% flowering was observed in the unfertilized treatment and the earliest 50% flowering was observed in the biotreatment. In addition, the inorganic fertilizer application showed the earliest 50% flowering (18.33 days) in the third cutting, and 50% SM application showed the longest 50% flowering day (59.33 day) in the first cutting. Among the fertilizers applied in this study, the earliest mean 50% flowering (32.33 days) occurred with biofertilizer alone, indicating its positive effect. It is thought that the earlier 50% flowering observed in the savory plants inoculated with biofertilizer may be due to the synthesizing of some plant growth-promoting substances. As a key nitrogen-fixing bacterium in the rhizosphere, azotobacter helps roots absorb nutrients by fixing atmospheric nitrogen into ammonium ions, increasing phosphorus availability during the flowering process of plants and reducing soil pH [33]. So, biotreatment has an indirect effect on flowering day, affecting hormone synthesis and photosynthetic availability.

Moreover, the reduction in days to flowering likely resulted from enhanced soil health, improved water retention, and increased beneficial microorganisms under combined sheep manure and biofertilizer treatment [34]. When our study was compared with the literature, Hadian et al. [35] reported that the 50% flowering days of different *S. hortensis* L. genotypes varied between 43 and 65 days. It is thought that the differences in the values obtained result from fertilizer type and ecological conditions. On the other hand, in agreement with our results, Pank et al. [36] reported that the 50% flowering days of *S. hortensis* genotypes varied between 29 and 51 days.

Plant height was determined three times before each sampling by measuring the height of 10 randomly selected plants per plot from the soil to the top of the plant, obtaining a mean value for each plot. As shown in Table 4, mean plant height values were found to be statistically insignificant in biofertilizer and sheep manure treatments but statistically significant in terms of second cutting time (*p* < 0.05). According to the treatments, the mean values varied between 26.80 and 30.10 cm. When evaluated according to the cutting time, the highest value was seen in the first cutting of 50% SM (40.93 cm) treatment, followed by unfertilized (39.17 cm) treatment in the first cutting and full SM (39.13 cm) treatment in the first cutting according to the observed tendencies. The lowest plant height was observed in the third cutting of unfertilized (14.97 cm) treatment, followed by the third cutting of 50% SM + Bio (16.20 cm) treatment and third cutting of 50% SM (17.23 cm) treatment according to the observed tendencies. The plant height values showed differences among the treatments depending on the cuttings. Plant height values at later harvests showed a regular decrease among all treatments, with the highest values in the first cutting. Sheep manure treatments (50% SM and full SM) showed higher values compared to biofertilizer alone, unfertilized and inorganic treatments. Further, 50% SM had the highest plant height in the first and second cuts, while full SM had the maximum height in the third cut. So, the treatment of full and 50% SM manure was superior to inoculation with azotobacter treatment.

In this study, the mean values of the plant branch number varied between 12.13 and 14.80. It is obvious from the data in Table 4 that the highest rate of branching was obtained in savory plants inoculated with the Bio and in the presence of full SM. However, the lowest rate of branching was obtained in unfertilized treatment. When evaluated in terms of cutting time, the highest branch number value was found in the first cutting of Bio + full SM (25.93 number), followed by the first cutting of sole azotobacter (24.10 number), and the first cutting of 50% SM (23.77 cm) treatment. The lowest branch number was determined in the third cutting of unfertilized (6.48 number), followed by third cutting of 50% SM + Bio (7.40 number) and third cutting of unfertilized (7.40 number) treatments according to the observed tendencies. Comparing the treatments, full SM + Bio and sole Bio treatment had positive effects on the branch number of savory in the first and second cuttings. Significant differences were found in the first and second cuttings depending on the treatments. Full SM + Bio and Bio treatments increased the branch number values by 19.33% and 10.91% compared to unfertilized treatment, respectively. In the second harvest, the Bio and full SM + Bio treatments increased the branch number values with 26.07% and 18.73%, respectively.

Sheep manure and biofertilizer treatments increased the plant height and number of branches in savory plants compared to the unfertilized control. This improvement can be attributed to the role of sheep manure and biofertilizers in promoting plant growth by supplying essential nutrients, enhancing soil properties and microbial activity [37,38]. Therefore, the use of sheep manure and Bio treatment in savory will have positive effects on plant height and branch number. Many investigations reported that treatment of azotobacter fertilization alone or with other fertilizers increased vegetative growth (plant height, number of branches and fresh and dry herb weights), nitrogen, phosphorus, and potassium levels in the tissues of medicinal plants [39].In earlier studies on *S. hortensis* L., it was reported that plant height changed between 23.5–39.9 cm [40], 23.73–30.02 cm [41] and 32.0–44.70 cm [42] under different ecological conditions. The present results comply with the mentioned results with respect to plant height. In this study, the number of plant branches’ data corroborate those reported by Aşcı [43] (20.4–25 number) and Çeri [42] (16.20–22.73) unit/plant; however, these data are much higher than those of Tansı and Tonçer [41] (5.36–7.98 number). The variation in branch number among cultivated plants is influenced by genotype, environmental conditions, and applied treatments.

### 3.2. Fresh and Dry Herb Weights

The fresh herb weight values of the savory were not significantly affected by the different fertilizers, according to statistical analysis of the data, except second cut (Table 5). In this study, total fresh herb values varied between 10.77 and 16.34 t/ha. The treatment of 50% SM recorded the highest total fresh plant weight, but the lowest total fresh herb weight was obtained in Bio treatment according to the observed tendencies. In terms of cutting time, the highest fresh herb weight was determined in the first cutting of 50% SM (10.19 t/ha) treatment, followed by the first cutting of the IO (7.31 t/ha) treatments according to the observed tendencies. The lowest fresh herb weight was found in the third cutting of 50% SM + Bio (1.49 t/ha), followed by the third cutting of full SM + Bio (1.55 t/ha) and the third cutting of Bio (1.56 t/ha) treatments according to the observed tendencies.

As can be seen from Table 5, the total dry herb weight values of savory fertilizer treatments were found to be statistically significant at the level of 0.05% (*p* < 0.05). The highest weight of dry herbs in terms of cutting time was determined in the first cutting of 50% SM (2.01 t/ha) treatment, followed by the first cutting of full SM (1.58 t/ha) and the first cutting of inorganic (1.40 t/ha) treatments according to the observed tendencies. The lowest dry herb weight value was found in the third cutting of 50% SM + Bio (0.32 t/ha) treatment, followed by the third cutting of Bio (0.35 t/ha) treatments according to the observed tendencies. Total dry herb values were highest in 50% SM (3.18 t/ha) treatment, followed by full SM (2.61 t/ha) and inorganic (2.33 t/ha) treatment. The lowest total dry weight value was obtained from unfertilized (1.99 t/ha) treatment, followed by Bio (2.02 t/ha) and full SM + Bio (2.05 t/ha) treatments. However, plants that were applied with full and 50% SM gave higher fresh and dry yields than those inoculated with biofertilizer, which may be attributed to the high organic matter and macro-element content of the soil. In addition, sheep manure improved the growth, and the yield of growth and development of plants is due to the humic acids and micro- and macronutrients presented in SM (Table 2). Furthermore, the findings suggest that to achieve high savory yield, the treatment of biological fertilizer alone is insufficient. Although biofertilizers can enhance nutrient availability and stimulate root growth through microbial activity, their sole treatment may not meet the immediate nutrient demands of fast-growing medicinal plants. This is primarily because biofertilizers release nutrients gradually, which may not fulfill peak nutrient requirements, especially under intensive cultivation systems. Thus, for optimal biomass accumulation, biofertilizers perform better when combined with other soil amendments [44,45].

Abd-Allah [46] reported that the combination of full dose of NPK + biofertilizer increased plant height, fresh and dry weights of *S. hortensis* L. In a study by Bakhtiari et al. [47], which evaluated the effects of combined inorganic (NPK), organic (vermicompost-VC) and biofertilizers (Thiobacillus, *Glomus mosseae*) on medicinal savory, optimum growth was observed with VC + NPK treatment. In a study investigating the effects of chitosan and different doses of biofertilizer EM treatment on the yield and quality characteristics of savory plants, it was stated that the plants inoculated with biofertilizer and chitosan spray increased growth and herb yield [17]. Mosapour and Feizian [48] reported that the main effect of the treatment of sheep manure biochar (0, 1 and 2% by weight) from soil and humic acid from leaves (0, 200 and 400 mg/L in three stages) on fresh and dry weight of savory shoot, fresh and dry weight of root, and stem diameter was positive in greenhouse conditions, and the highest fresh weight, shoot dry weight, root fresh weight values were obtained in 400 mg/L humic acid + 2% biochar treatment.

In addition, when the studies were examined, our fresh herb yield results (10.76–16.34 t/ha) were found to be higher than the reported values such as Kızıl and Tonçer [49] (3.90–5.96 t/ha) and Aşcı [43] (7.91 t/ha) in first year and (10.85 t/ha) in the second year. However, in line with our result, Çeri [42] reported that the green herb yield varied from 10.46 to 20.36 t/ha.

The dry herb yield (1.99–3.18 t/ha) obtained in the current study was comparable to that of the one reported in a study by Aşcı [43], who found that the dry herb yield of savory ranged from 3.45 to 4.56 t/ha in different years. In a study conducted by Çeri [42], the dry herb yield of the savory plant was reported as between 1.26 and 4.24 t/ha in different growth periods. The co-application of sheep manure and biofertilizer exerts multiple beneficial effects on soil and plant performance. Specifically, it contributes to the improvement of soil physical, chemical, and biological properties. Moreover, it enhances the availability and uptake of both macro- and micronutrients in plants. These amendments also influence several physical and biochemical processes within the savory plant system, ultimately resulting in significantly increased dry weight yield [50]. Thus, these differences may be responsible for the effects of sheep manure and biofertilizers on soil properties, as well as genetic and ecological factors. In the third cutting of summer savory, a noticeable decline in fresh herb weight was detected, commonly attributed to environmental changes such as lower temperatures and shorter photoperiods toward the end of the growing season. This reduction in biomass corresponds with a natural senescence and reduced vegetative growth potential. Consistent with these findings, Khalid [51] reported that cutting time has significant impacts on the fresh herb yield in medicinal plants. In general, the first cutting yields the highest fresh weight due to optimal vegetative growth under favorable environmental conditions, such as more extended daylight hours and moderate temperatures. Moreover, Kızıl et al. [52] noted that the second cutting often exhibits a moderate decline in fresh biomass, as the plant regrowth capacity may be affected by prior cutting stress and changing climatic conditions. A substantial reduction in fresh herb weight is typically observed by the third cutting, mainly attributed to the cumulative cutting stress, reduced photosynthetic efficiency, shorter photoperiods, and lower late-season temperatures, which collectively limit vegetative regrowth.

### 3.3. Essential Oil Contents

The mean values of essential oil (EO) obtained in this study varied between 1.04 and 1.43% *v*/*w*. Although no statistically significant differences were found among the different cuttings except for the third cutting, the highest essential oil content was obtained in the second cutting with inorganic (2.01% *v*/*w*) treatment, followed by the second cutting with 50% SM (1.80% *v*/*w*) treatment and the first cutting with inorganic (1.78% *v*/*w*) treatment according to the observed tendencies (Table 6). The lowest essential oil content was obtained in the third cutting with 50% SM (0.26% *v*/*w*) treatment, followed by the third cutting with the inorganic (0.51% *v*/*w*) treatment and the third cutting with the unfertilized (0.52% *v*/*w*) treatment.

In general, higher essential oil content values were achieved in the second cutting. This pattern is likely due to increasing temperatures during the second cutting (Figure 1). This observation aligns with previous findings, suggesting that drought stress induces a physiological response characterized by enhanced synthesis of secondary metabolites, which, in turn, contributes to increased essential oil content [53]. In addition, the levels of secondary metabolites in plants vary in response to seasonal changes, diurnal cycles, and climate conditions [54]. The effect of azotobacter on the essential oil content of savory herb was minimal. The unfertilized treatment exhibited the highest EO content, suggesting that nutrient limitations may enhance the biosynthesis of secondary metabolites as a plant stress response mechanism. Many studies have shown that nutrient limitation in plants leads to increased accumulation of secondary metabolites, particularly terpenoids and phenolic compounds. In line with the Carbon–Nutrient Balance Hypothesis (CNBH), a reduction in nitrogen availability results in excess carbon, which is subsequently allocated to the biosynthesis of carbon-based secondary metabolites rather than primary growth processes, thereby promoting essential oil production [55,56]. Furthermore, EO content was higher in inorganic fertilizer-treated plots compared to those treated with sheep manure. Hence, SM + Bio treatments reduced the essential oil content due to enhancing yield and alleviating stress conditions [57,58].

Our results are compatible with the essential oil content of savory plants grown under biofertilizer treatment by Amani et al. [59] and different levels of inorganic fertilizer by Najafian and Zahedifar [60]. Abd-Allah [46] reported that the highest oil yield resulted from the plants fertilized with the full dose of NPK + biofertilizer, but the lowest content was produced from treated plants with biofertilizer alone. In another study, it was reported that chitosan and biofertilizer EM treatment increased the amount of essential oil in savory plants [17]. In addition, Fathi et al. [61] reported that the Uzbekistan accession exhibited the highest essential oil content (0.75% *v*/*w*) among the different savory accessions. These differences can be attributed to the biofertilizer treatments, genetic differences, soil and ecological factors. Ecological conditions, such as light quality and intensity, photoperiodic effects, temperature, water, soil, altitude, and wind, may significantly affect the essential oil content and secondary plant metabolites [62]. In addition, chemical stress (salinity, pH, fertilization, chemical composition and toxins) affects the quality and quantity of essential oil of medicinal and aromatic plants as environmental stress sources [63].

### 3.4. Essential Oil Compositions (% v/v)

As shown in Table 7 and Table 8, seven major components (carvacrol, thymol, γ-terpinene, α-terpinene, cymol, α-bisabolene, and α-phellandrene) were dominant, representing 29.28–54.58% *v*/*v* of the total essential oil compounds. The main components, except for carvacrol, were significantly enhanced due to sheep manure + Bio treatments relative to the unfertilized plots.

Carvacrol value means among the treatments varied between 7.56 and 20.95% *v*/*v*. In the comparison of the cuttings, the highest carvacrol content was obtained in the unfertilized (42.54% *v*/*v*) treatment in the third cutting, followed by inorganic (26.74% *v*/*v*) treatment in the third cutting and Bio + full SM (22.98% *v*/*v*) treatment in the third cutting (Table 7). Considering the total cutting data, the increase in the carvacrol in the first and second cuttings was less than the third cutting. The lowest carvacrol content was observed in the full SM treatment (0.18% *v*/*v*), followed by the 50% SM treatment (2.51% *v*/*v*) and the Bio + 50% SM treatment (4.42% *v*/*v*) in the second cuttings. Moreover, the lowest carvacrol content was found in the presence of full-dose sheep manure.

The mean thymol values among different fertilizer treatments varied between 0.36 and 5.68% *v*/*v*. In the comparison of cuttings, the highest thymol content was obtained in the full SM (16.09% *v*/*v*) treatment in the second cutting, followed by the 50% SM (8.60% *v*/*v*) treatment in the second cutting and the 50% SM (4.39% *v*/*v*) treatment in the third cutting (Table 7). The lowest thymol content was obtained in the third cutting with 50% SM + Bio (0.05% *v*/*v*) treatment, followed by the third cutting with full SM (0.11% *v*/*v*) and the second cutting with Bio (0.12% *v*/*v*), and the first cutting with 50% SM (0.12% *v*/*v*) treatments with sheep manure.

It was also found that full and 50% doses of sheep manure resulted in a significantly higher thymol content in savory plants compared to both the Bio and the unfertilized treatments. In addition, the combined treatment of Bio and sheep manures showed the lowest thymol content.

The mean γ-terpinene values obtained in this study varied between 6.52 and 10.38% *v*/*v*. In the comparison of the cuttings, the highest γ-terpinene content was obtained in the inorganic (16.05% *v*/*v*) treatment of the third cutting, followed by the treatment of full SM + Bio (15.38% *v*/*v*) in the third cutting and the treatment of inorganic (9.00% *v*/*v*) in the first cutting (Table 7). The lowest γ-terpinene content was obtained in the treatment of 50% SM + Bio (3.18% *v*/*v*) in the second cutting, followed by the treatment of 50% SM (4.83% *v*/*v*) in the first cutting and the treatment of 50% SM (5.79% *v*/*v*) in the second cutting.

The mean values of α-terpinene varied between 3.48 and 7.22% *v*/*v*. In the comparison of the cuttings, the highest α-terpinene content was obtained in the third cutting of the full SM + Bio (10.02% *v*/*v*) treatment, followed by the first cutting of the full SM + Bio (6.14% *v*/*v*) treatment and the inorganic (5.71% *v*/*v*) treatment of the first cutting. The lowest α-terpinene content was obtained in the unfertilized (1.32% *v*/*v*) treatment of the third cutting, followed by the first cutting of the 50% SM (1.42% *v*/*v*) treatment and the Bio (2.01% *v*/*v*) treatment of the first cutting. The results showed that the combination of Bio and 50% of sheep manure produced highest α-terpinene content compared to other treatments. So, the combination of SM + Bio treatment was significantly higher than other treatments.

In our study, we noticed a decrease in thymol levels when Bio and SM alone were applied, while γ-terpinene showed a notable increase in response to Bio + SM treatments. This observation aligns with the findings of Sarmoum et al. [64] and Raffo et al. [65], highlighting that the biosynthesis of terpenoids happens through different pathways. Similarly, Kachur and Suntres [66] reported that thymol and carvacrol are positional-isomers, sharing a similar chemical structure with a hydroxyl group and an isopropyl group attached to a benzene ring, while terpinenes, which have a similar chemical structure to α-phellandrene, are isomeric hydrocarbons differing in the location of their carbon–carbon double bonds in their chemical skeletons. Although the biosynthesis of secondary metabolites is genetically controlled, it is strongly affected by environmental influences [67]. In addition, the use of organic and biofertilizers can also increase the EOC by enhancing the uptake of P and N, which are the major prerequisites for the primary and secondary metabolism in most medicinal plants [68,69]. Similar to our results, Edris et al. [70] found that the relative percentage of certain constituents of marjoram essential oil was affected by fertilization type and level. Gharib et al. [71] reported that the interaction treatment of nitrogen-fixer strains + compost treatment in *Majorana hortensis* had the highest percent of γ-terpinene, α-terpinene and α-phellandren. Our results were in harmony with those obtained by Gharib et al. [71], Amzallag et al. [68], and Bernstein et al. [69].

Additionally, it was observed that the contents of α-carvacrol, thymol, and γ-terpinene increased in the third harvests. Likewise, seasonal changes occurring over the cutting period affect the content and component of essential oil [72]. Climatic changes occurring over the approximately 4-month period difference between the first and last cuttings influenced essential oil component. In the third cutting, decreasing temperatures correlated with increases in key components. Similarly, many studies reported that carvacrol and thymol contents decrease under high-temperature conditions, likely due to the thermal degradation of these phenolic monoterpenes or altered biosynthetic enzyme activity. Additionally, elevated temperatures may inhibit their biosynthesis pathways, resulting in reduced accumulation in aromatic plants, such as thyme and oregano [73,74]. Therefore, a comparison of the experimental data with literature values revealed that current results were consistent with those reported by [73,74].

In previous studies, the essential oil of summer savory was also found to have good antioxidant activity, owing to the presence of the dominating oxygenated monoterpenes, thymol and carvacrol [75]. Therefore, it is considered that the antioxidant activities in savory extracts are related to the high content of these components. Other important terpenoids found in the essential oil are γ-terpinene, myrcene, p-cymene, linalool, β-caryophyllene, α-pinene, and some derivatives [76,77].

In contrast, Çeri [42] reported that carvacrol (55.94–65.24% *v*/*v*), γ-terpinene (21.67–27.00% *v*/*v*) and p-cymene (9.47–16.21% *v*/*v*) were prominent as the main components of leaf essential oil of *S. hortensis*. The difference in the results from our study may be due to differences in chemotypes, as well as the part of the plant utilized [72].

In addition, the obtained results were in agreement with findings mentioned by Abd-Allah [46], who reported that the highest component of savory oil was carvacrol, and the treatment of biofertilizer alone decreased the carvacrol percentage. Similarly, Esmaielpour et al. [78] obtained the highest carvacrol content (62.90% *v*/*v*) when 50% washed mushroom compost was applied. Our results are consistent with the results obtained from the study conducted by Hadi et al. [79] with biological nitrogen fertilizers and worm compost.

In contrast, Ghojavand et al. [80] reported that the thymol content of plants inoculated with any of the biofertilizers, alone or together, increased statistically compared to the unfertilized treatment. Toaima et al. [17] determined that the main components of the essential oil of savory plants were γ-terpinene and carvacrol, and the highest levels of γ-terpinene and carvacrol were detected in oils obtained from plants treated with chitosan and biofertilizer. Fathi et al. [61] noticed that the values of carvacrol, thymol and γ-terpinene in different origins of savory oils were found from 0.004 to 28.07% *v*/*v*, from 1.95 to 41.13% *v*/*v*, and from 2.15 to 84.03% *v*/*v*, respectively. Dardioti et al. [81] investigated the diversity of *Satureja pilosa* essential oil compounds and reported that the thymol, *p*-cymene, and carvacrol amounts had the highest variation.

The mean values of cymol varied between 2.12 and 5.90% *v*/*v*. In the comparison of the cuttings, the highest cymol content was obtained in the inorganic treatment of the third cutting (9.49% *v*/*v*), followed by the inorganic treatment of the first cutting (5.06% *v*/*v*) and the 50% SM treatment of the second cutting (5.05% *v*/*v*). The lowest cymol content was obtained in the unfertilized treatment of the third cutting (0.04% *v*/*v*).

So, inorganic fertilizer gave savory plants a high cymol content, in comparison with inoculation with the sheep manure and unfertilized treatment (Table 8). In addition, full and 50% doses of sheep manure increased the mean cymol content compared to the sheep manure + Bio and unfertilized treatment. Ruberto and Baratta [82] reported that α-terpinene and γ-terpinene (isomeric monoterpenes) aromatic compounds have powerful antioxidant properties. Thus, these valuable aromatic compounds observed in the savory plant are very important because they prevent the oxidation of other compounds by preventing or delaying the beginning or proliferation of oxidative chain reactions [83]

The mean α-bisabolene values among the treatments ranged from 2.97% *v*/*v* to 5.28% *v*/*v*. In the comparison of the cuttings, the highest α-bisabolene content was obtained in the inorganic (7.09% *v*/*v*) treatment in the third cutting, followed by the unfertilized (5.29% *v*/*v*) treatment in the third cutting and the Bio + full SM (4.82% *v*/*v*) treatment in the third cutting (Table 8). The lowest α-bisabolene content was obtained in the full SM (0.79% *v*/*v*) treatment in the second cutting, followed by the Bio (1.03% *v*/*v*) treatment in the second cutting and the 50% SM (2.09% *v*/*v*) treatment in the first cutting. Rioba et al. [84] reported that high phosphorus applied to the *Chamomilla recutita* plant increased the α-bisabolol content. This result is consistent with our study; high α-bisabolol was obtained from the treatment of sheep manure with high phosphorus content. the α-bisabolene compound is an organic compound with a therapeutic effect and acts as an antioxidant; like the most important antioxidant, vitamin E, it can also be used as an alternative for vitamin E. Therefore, since this compound is used as an effective ligand alternative to vitamin E, the savory plant can be used to prevent or treat some different conditions such as cancer, eye disorders and others [85]. Therefore, savory herbs were found to be suitable for the production of α-bisabolene as an antioxidant vitamin E in food, and it would also have economic value for the grower.

The mean α-phellandrene values obtained in this study ranged from 1.86% *v*/*v* to 6.44% *v*/*v*. In different cuttings, the highest α-phellandrene content was obtained in 50% SM + Bio (9.76% *v*/*v*) treatment in the second cutting, followed by Bio (7.18% *v*/*v*) treatment in the first cutting and unfertilized (7.16% *v*/*v*) treatment in the second cutting. The lowest α-phellandrene content was obtained with unfertilized (0.03% *v*/*v*) treatment in the third cutting, followed by Bio + full SM (0.15% *v*/*v*) treatment in the third cutting and inorganic (0.17% *v*/*v*) treatment in the third cutting.

Alpha-phellandrene and oils rich in this molecule have been proven to be potential biopesticides, larvicides and insect repellents, while recent studies have reported antimicrobial and antitumoral effects of alpha-phellandrene as a pure compound and in synergy with drugs [86]. So, α-phellandrene was found in higher concentrations in Bio + 50% SM treatment compared to other treatments. Therefore, Bio + 50% SM treatment may play a significant role in enhancing antimicrobial and antitumoral effects and healthy living.

Totally, thirty-four essential oil compositions were detected, and twenty-seven essential oil compositions were found as minor. The minor essential oil compositions values of the savory grown under different-dose SM + biofertilizer treatments are given in Tables S2–S6.

### 3.5. Total Phenolic, Total Flavonoid and Antioxidant Activities

#### 3.5.1. Total Phenolic Content (TPC)

Mean TPC values among different fertilizer treatments varied between 51.28 and 64.46 mg GAE/g DW. In the comparison of the cuttings, the highest TPC was obtained in the third cutting with 50% SM + Bio (101 mg GAE/g DW) treatment, followed by the third cutting with 50% SM (96.87 mg GAE/g DW) treatment and the third cutting with Bio (58.64 mg GAE/g DW) treatment (Table 9). The lowest TPC was obtained in unfertilized (29.56 mg GAE/g DW) treatment in the second cutting, followed by Bio (30.36 mg GAE/g DW) treatment in the first cutting and inorganic (33.31 mg GAE/g DW) treatment in the first cutting. In particular, sheep manure and sheep manure + azotobacter treatments increased the total phenolic contents. As in DPPH activity, the highest phenolic content was obtained from the Bio + 50% sheep manure treatment among the cuttings.

As seen in Table 9 and Figure 1, there is a relationship between phenolic compounds and temperature. The temperature decline observed in September and October, corresponding with the third harvest, was positively correlated with an enhanced accumulation of total phenolic contents.

When studies on this subject were examined, the highest biosynthesis of phenolics in *Lavandula viridis* cultures was observed at 15 °C [87]. Similarly, cultures of *Ajuga bracteosa*, another plant belonging to the Lamiaceae family, were exposed to 10, 15, 20, 25, and 30 °C, and the maximum phenolic contents were reached at 15 °C [88].

In our study, the total phenolic content (TPC) increased with SM and Bio treatments, likely due to the high Cu, Pb, Fe and Zn concentrations in SM [89]. Because the micronutrient Cu activates the phenylalanine ammonia lyase (PAL) enzyme activity pathway, enhancing phenolic compound production in plants, this demonstrates the positive correlation between soil Cu and tannins/flavonoids levels [90]. According to Jin et al. [91], phenolic compounds can complex with Fe^3+^ and facilitate its mobilization between tissues. They may also participate in reducing Fe^3+^ to Fe^2+^, assisting reductase-type enzymes. Consistent with our findings, the TPC determined in *Guadua angustifolia* Kunth plants under organic and chemical fertilizer treatments was significantly higher compared to the control groups [92]. Another study reported that vermicompost positively influenced TPC levels in *Berberis integerrima* Bunge plants under cadmium stress [93].

Our result was higher than those reported by Alizadeh et al. [94], who found that the total phenolic content varied between 23.58 and 24. 52 mg GAE/g DW. Similarly, Dorman and Hiltunen [95] estimated the total extractable phenolic contents of the savory crude extract and n-butanol and water fractions as 27.0 and 67.2 mg GAE/g, respectively. Furthermore, Taie et al. [96] reported that the total phenolic and flavonoid contents in plants grown with organic fertilizer were higher than those grown with inorganic fertilizer

Yaldız and Çamlıca [3] determined that the phenolic content of savory plant varied between 746 and 1087 µM at different cutting times, and the highest total phenolic content was determined at the full bloom stage of the plant.

Brown [97] revealed that *Azotobacter paspali* can release IAA in the medium, and Reda et al. [98] reported that growth regulators increase total phenolic content in thyme. The differences between the results of the current study and those of earlier studies may be due to the use of different extracts for analyses, environmental and genetic factors, chemo-types and the nutritional status of the plants, as well as other factors that may have an influence on the antioxidant activity and total phenolic content. Several studies have suggested relationships between antioxidant activity and phenolic contents of plants [46,47,99]. Similarly, in the current study, we found a positive correlation between the total phenolic content and antioxidant activity in all plant extracts.

#### 3.5.2. Total Flavonoid Content (TFC)

As can be seen from Table 9, the TFC values were found to be statistically significant at the level of 0.05% in terms of treatments and cutting time (*p* < 0.05). Mean values of TFC among the treatments varied between 22.32 and 33.36 mg QE/g DW. In the comparison of the cuttings, the highest TFC was obtained in the third cutting with 50% SM + Bio (45.97 mg QE/g DW) treatment, followed by the unfertilized (45.61 mg QE/g DW) treatment in the first cutting and the full SM (41.66 mg QE/g DW) treatment in the third cutting (Table 9). The lowest TFC was obtained in the second cutting with 50% SM (10.75 mg QE/g DW) treatment, followed by the unfertilized (11.94 mg QE/g DW) treatment in the second cutting and the inorganic (18.10 mg QE/g DW) treatment in the third cutting. When cutting times were evaluated together, the highest TFC was obtained from the third cutting. Also, the combinations of sheep manure doses + Bio treatments increased TFC in the three cuttings.

The seasonal decreases in flavonoid contents may be attributed to their conversion into insoluble cell wall components or their transformation into oligomeric and polymeric compounds such as tannins or lignans [100]. Notably, low temperature significantly increases flavonoid content in various plant species, the abundance of enzymes involved in flavonoid biosynthesis and the expression of genes in this pathway [101,102].

In our study, as in TPC, the highest TFC contents were observed in sheep manure and Bio treatments. Among the micronutrients found in sheep manure, Zn and Cu, in particular, increased the amount of TFC. Both Cu and Zn are essential micronutrients for plants, and they can act as elicitors, inducing stress mechanisms that stimulate the production of bioactive secondary metabolites [103,104].

Our study’s results align with those reported by El-Leithy et al. [105], who found that nitrogen fertilization enhanced the TFC in *S. hortensis* L. Likewise, in our investigation, an increase in flavonoid contents was observed when using the full SM treatment compared to the unfertilized treatment.

#### 3.5.3. DPPH Free Radical Scavenging Activity

Among different fertilizer treatments, DPPH mean values varied between 48.46 and 28.62%. The combined treatment between Bio and sheep manure was effective in improving the DPPH mean values of savory, and the highest values were obtained with the combined Bio + full SM treatment (Table 10). Thus, the inoculation of Bio + sheep manure may encourage the production of biologically active substances, such as phytohormones, amino acids, and water-soluble vitamins. Some activating hormones, which play an essential role in biofertilization, may increase the contents of IAA, cytokinins and GA [106]. In the comparison of cuttings, the highest DPPH value was obtained in the second cutting of the mixture of 50% SM + Bio (60.86%) treatment, followed by the second cutting of the inorganic (54.93%) treatment and the second cutting of Bio + full SM (53.85%) treatment. The lowest DPPH value was obtained in the third cutting with full SM (9.44%) treatment, followed by the third cutting of 50% SM + Bio (12.29%) treatment and the third cutting with Bio (17.46%) treatment. So, the second cutting exhibited significantly higher antioxidant capacity values compared to the first and third cuttings. This can be attributed to the temperature increase observed in August. It is likely that temperatures approaching 30 °C contributed to enhanced bacterial activity ([107], Figure 1). Furthermore, the mixed Bio + sheep manure had pronounced enhancing effects on the increase in DPPH levels in the savory herbs. This is due to the high nitrogen content in sheep manure; this shows that certain doses of sheep manure together with biofertilizer are sufficient to improve antioxidant activity (Table 2).

Similar to our study, *Perilla frutescens* (Lamiaceae) was grown ex vitro under different temperature regimes (15/10, 20/15, 25/20, 30/25, and 35/30 °C) and showed the highest DPPH scavenging capacity at 35/30 °C [108]. Furthermore, *Melissa officinalis* (Lamiaceae) confirmed higher antioxidant capacity at higher temperatures [109].

According to the literature, carvacrol and thymol have been shown to contribute significantly to the antioxidant power of plants. In our study, unfertilized and full SM + Bio treatments containing high carvacrol content had high antioxidant contents (Table 10). Therefore, the strong antioxidant activity of savory, measured using the DPPH method, can be attributed to its high carvacrol content (42.54% *v*/*v*). Similar to our results, carvacrol has been reported to have better antioxidant activity than thymol, regardless of the assessment method including DPPH [110,111,112].

Carvacrol and thymol are known to possess many interesting biological activities, including antimicrobial, antioxidant, anti-inflammatory, and analgesic properties [113,114]. These properties make them promising compounds for numerous pharmaceutical and cosmetic treatments [115].

When our study was compared with the literature, Exarchou et al. [116] reported 95.8% and 33.00% DPPH activity of ethanolic and acetone extract in savory. In a similar study, Alizadeh et al. [94] investigated the antioxidant activity of *S. hortensis* extracts using a DPPH free radical scavenging assay, and the IC_50_ values ranged from 8.45 to 8.60 μg/mL. In a study conducted by Najafian and Zahedifar [60], it was determined that different levels of inorganic fertilizer could significantly increase the antioxidant activity of *Satureja hortensis* L. Yaldız and Çamlıca [3] recorded antioxidant activity between 6.65 and 16.10 µM catechin equivalent at different cutting times of savory plants and determined that the highest antioxidant activity was at the full bloom stage of the plant.

The presence of phenolic acids such as rosmarinic acid derivatives has been detected in *S. hortensis* [117], and it has been shown that these polyphenols have antioxidant activity in different test systems [118]. Therefore, the activity observed in the DPPH experiment may be related to the presence of rosmarinic acid derivatives in the extract. The differences between the results of the current study and those of earlier studies may be due to the use of sheep manure and biofertilizer, environmental and genetic factors, different extraction methods of the plants as well as other factors that may have an influence on the antioxidant activity and total phenolic content.

#### 3.5.4. Iron-Reducing Antioxidant Power (FRAP)

Mean FRAP values among the treatments ranged from 35.27 mg TE/g DW to 57.39 mg TE/g DW. In the comparison of the cuttings, the highest FRAP content was obtained in the full SM (69.64 mg TE/g DW) treatment in the first cutting, followed by the unfertilized (63.67 mg TE/g DW) treatment in the first cutting and the full SM (58.64 mg TE/g DW) treatment in the second cutting (Table 10). The lowest FRAP content was obtained in the unfertilized (25.13 mg TE/g DW) treatment in the second cutting, followed by the treatment of Bio + 50% SM (27.28 mg TE/g DW) in the second cutting and the treatment of 50% SM (28.06 mg TE/g DW) in the third cutting. As seen in the Table 10, sheep manure and Bio treatments increased the FRAP values.

Dorman and Hiltunen [95] found that the non-polar hexane-soluble fraction had a low ascorbic acid equivalent (AscAE) value of 37.1 μmol ascorbic acid per gram of extract. Similarly, the n-butanol (n-BuOH) fraction had a similar value of 27.0 μmol ascorbic acid per gram of extract. In contrast, the water (H_2_O) fraction exhibited a better reducing potency, with an AscAE value of 67.2 μmol ascorbic acid per gram of extract. However, the polar and highly polar fractions showed poor activity, with IC_50_ values of 5.49 mg/mL and 8.82 mg/mL, respectively. Also, Mašković et al. [119] reported that *S. hortensis* extract showed high antioxidant activity by measuring antioxidant properties with different methods, such as FRAP, ABTS, and DPPH. So, savory extract has a good source of antioxidants, and this plant herb is used to enhance items in the food, feed, and pharmacological industries due to its antioxidant properties.

### 3.6. Principal Coordinate Analysis (PCA)

**Figure 2:**
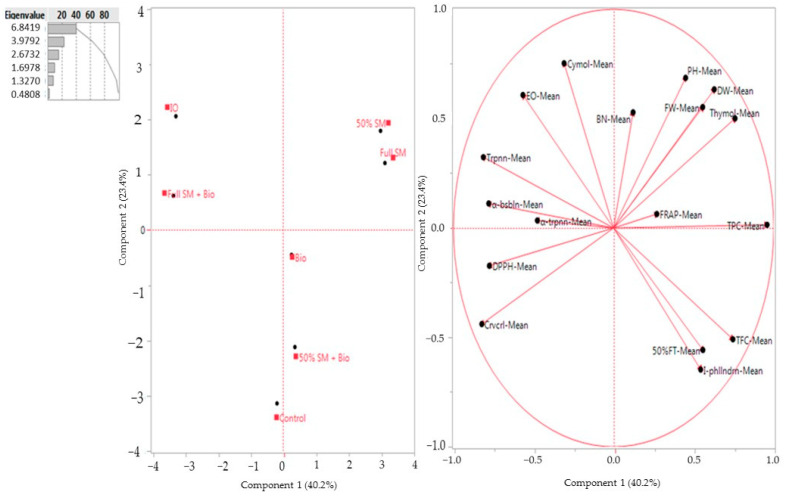
PCA analysis results of the means of the examined properties according to cut results. SM: sheep manure, Bio: biofertilizer, IO: inorganic fertilizer, 50% FT: 50% flowering time, PH: plant height, BN: branch number, FW: fresh weight, DW: dry weight, EO: essential oil, DPPH: 1,1-diphenyl-2-picrylhydrazyl, FRAP: ferric-reducing antioxidant power, TPC: total phenolic content, TFC: total flavonoid content, I-phllndrn: α-phellandrene, α-trpnn: α-terpinen, α-bsbln: α-bisabolene, trpnn: Gama-terpinene, crvcrl: carvacrol.

A PCA was performed for the means of the morphological, yield, essential oil content, antioxidant activities and major essential oil composition properties assessed in this study, which were shaped by the investigated treatments and their significant interactions. The first two components accounted for 63.6% of the total variance (Figure 2). The two-dimensional component plot revealed an internal data structure that aligned with the experimental treatments (Figure 2). Samples were separated coherently along the PC1 based on the results, with plant height, fresh and dry herb weights, thymol content and total phenolic content in the positive PC1 plot area. Given the prominent contribution of the first component with 40.2% of the total variance, the results were associated with the largest linearly projected variance in the measured savory properties. Moreover, samples were much more distributed with the full SM and 50% SM treatments, indicating that the total common variance of the savory morphological and yield properties was restrained when plants grow with sheep manure treatments. The second PC (PC2) exhibited 23.4% of the total variance, and different variables had different percentages of contribution in total variability.

The results are shown in Figure 2, and the first two PCs revealed more than 63%. We obtained results for PCA compared to previous studies; the first two PCs were found with 75.83% (51.11% PC1 and 24.72% PC2) related to morphological and yield values of oregano [120]. According to treatments, 50% SM and full SM treatments showed positive effects on the plant height, fresh weight, dry weight and thymol contents based on the PCA results. It was noted that inorganic fertilizer and full SM + Bio treatments revealed a relationship with the essential oil contents and compositions, except thymol and I-phellandrene (Figure 2). In PC1, total fresh yield, γ-terpinene, as well as carvacrol were in PC1, and plant height, dry matter yield, total phenolic content, α- thujene, α-pinene, p-cymene and thymol contents were noted in PC2 [120]. In another study, Constantin et al. [121] conducted a study on the identification of the main physico-chemical properties affecting the quality of *Satureja hortensis* plant. It was determined that PC1 and PC2 account for 59% and 23% of the total variation.

### 3.7. Heatmap Analysis

**Figure 3:**
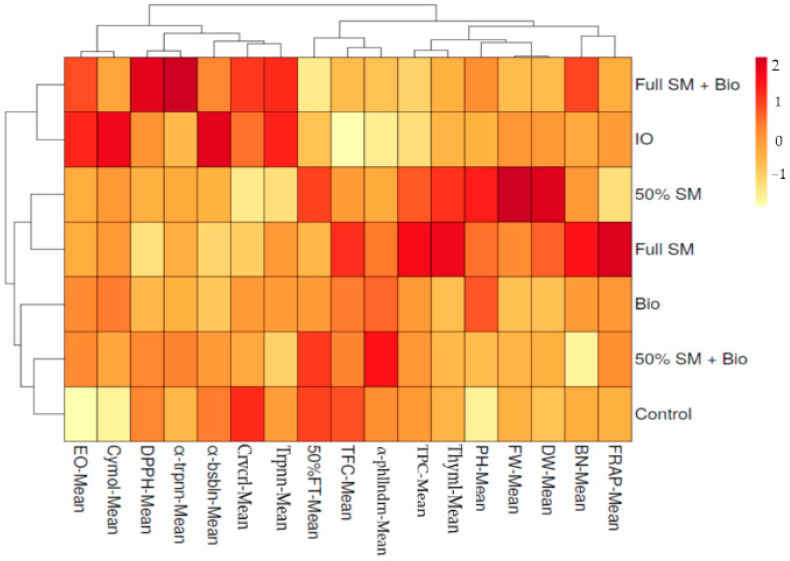
Relationship between the chemical properties means of savory plants. SM: sheep manure, Bio: biofertilizer, IO: inorganic fertilizer, 50% FT: 50% flowering time, PH: plant height, BN: branch number, FW: fresh weight, DW: dry weight, EO: essential oil, DPPH: 1,1-diphenyl-2-picrylhydrazyl, FRAP: ferric-reducing antioxidant power, TPC: total phenolic content, TFC: total flavonoid content, α-phllndrn: α-phellandrene, α-trpnn: α-terpinen, α-bsbln: α-bisabolene, trpnn: γ-terpinene, crvcrl: carvacrol.

A heatmap analysis of the cut mean values of the essential oil content, antioxidant activities and major essential oil compositions was conducted to achieve a graphical appraisal of the influences determined by the different treatment factors on savory plants (Figure 3). The heatmap output consisted of two clusters, cluster 1 sited along the top containing all the examined properties and cluster 2, located on the right side, comprising all treatment influencing this distribution. Cluster 1 presented two main clusters: the first one, on the bottom, grouped ten examined properties, mostly including antioxidant activities (except DPPH), all morphological and yield values with thymol and I-phellandrene essential oil compositions. This cluster showed two sub-groups, the first on the left, grouping 50% flowering day, total flavonoid content and α-phellandrene of cluster 1A, which were aggregated mainly on the basis of treatments of full SM + Bio and Bio. The second sub-group, on the right, included total phenolic content with morphological and yield values and thymol of cluster 1B, characterized by full SM, 50% SM, and inorganic fertilizer treatments (Figure 3). The main cluster 2 included seven properties, and this cluster was separated from other groups by the 50% SM, full SM and Bio treatments. The second main cluster, which included the remaining seven treatments, was divided into two sub-groups too. The first one, on the right, grouped all of the treatments of cluster 2, except full SM + Bio and inorganic fertilizer. These were aggregated on the basis of their tendentially higher values of applied treatments. The first group related to the treatments (full SM + Bio and inorganic fertilizer) was found by the main factors of the essential oil contents and γ-terpinene values. The second leading group showed differences, with 50% and full sheep manure treatments related to the essential oil contents, α-terpinene, carvacrol and cymol properties found in the same group.

The heatmap analysis revealed the main cluster according to the examined properties; many of the properties were found in the same cluster, except and DPPH and carvacrol properties. The obtained results agreed with Çamlıca and Yaldız [99], who reported that total phenolic and flavonoid content values were found in the same group in basil plant according to heatmap analysis.

## 4. Conclusions

As a result, some quality characteristics (essential oil ratio, essential oil components and antioxidant values) and herb yield of savory plant showed differences between the treatments. The results showed that 50% sheep manure yielded the highest total fresh and dry herb yield. According to data, the highest essential oil contents among treatments occurred in inorganic treatment. The highest carvacrol and α-terpinene rates among treatments were obtained in unfertilized treatment, while the highest thymol rate was obtained in full sheep manure treatment. The highest DPPH values were obtained with 50% SM + Bio treatment, and the highest FRAP content occurred in full SM treatment. Among treatments, the highest total phenolic and total flavonoid contents were obtained with 50% SM + Bio. PCA and heatmap analysis showed important results in treatments and examined properties. Treatments of full SM and 50% SM correlated with fresh and dry herb weight values in both analyses. Results may help reduce chemical fertilizer use, promote organic fertilizers, and support healthier, higher savory production. Therefore, to reduce the excessive organic/chemical fertilizer use, minimize production costs, improve soil structure, and increase savory quality/essential oils/antioxidant activity, reduced sheep manure with biofertilizer is recommended.

## Figures and Tables

**Table 1:** Treatment codes, description and rate per plot.

Treatment Code	Description	Doses of Treatment Per Plot
50% SM	Sheep manure (11.25 t/ha)	Sheep manure applied as bottom dressing at 11.25 t/ha
50% SM + Bio	50% sheep manure + azotobacter	Sheep manure applied at 11.25 t/ha with azotobacter inoculation
Bio	Azotobacter (*Azotobacter chroococcum* and *Azotobacter vinelandii*)	100 mL Vitormine plus drip was added in a 20 L water
Unfertilized	Control	No treatment bio/inorganic/sheep manure fertilizer
Full SM	Sheep manure (22.50 t/ha)	Sheep manure applied as bottom dressing at 22.50 t/ha
Full SM + Bio	Full sheep manure + azotobacter	Sheep manure applied at 22.50 t/ha with azotobacter inoculation
IO	Inorganic 100%	DAP fertilizer applied as bottom dressing at 40 kg/ha and Ammonium sulphate (NH_4_)_2_SO_4_ applied as bottom dressing at 30 kg/ha. Also, Ammonium sulphate (NH_4_)_2_SO_4_ applied as top dressing at 30 kg/ha.

**Table 2:** The chemical properties of used sheep manure in this study.

Properties	Unit	Analysis Method	Results
Moisture	%	A.O.A.C. 1995	6.13
Organic matter	%	TL 7.02.32 (Rev.4) (AOAC 967.03,04,05)	74.58
pH		1/10 Potentiometric	7.44
EC	dS/m	1/10 Potentiometric	4.14
Total nitrogen (N)	%	TL 7.02-02 (Rev:4) TS EN 15478	2.71
Total potassium (K)	%	ICP, EPA 3052	1.13
Total copper (Cu)	ppm	GPGDY EK-2 9.1/10.1	140.00
Total phosphorus (P)	%	ICP	0.33
Total calcium (Ca)	%	GPGDY EK-2 9.1/10.1	1.87
Total magnesium (Mg)	%	GPGDY EK-2 9.1/10.1	0.33
Total iron (Fe)	%	GPGDY EK-2 9.1/10.1	0.38
Total manganese (Mn)	ppm	GPGDY EK-2 9.1/10.1	340.00
Total zinc (Zn)	ppm	GPGDY EK-2 9.1/10.1	240.00

**Table 3:** 50% flowering days of the savory plants grown under different fertilizer treatments.

Treatments	1st Cut	2nd Cut	3rd Cut	Mean
50% SM	59.33 a	27.00 ab	22.33 cd	36.22 ab
Unfertilized	49.00 c	29.67 a	30.50 ab	36.39 a
Full SM	49.67 bc	27.00 ab	27.00 abc	34.56 abc
50% SM + Bio	48.33 c	29.33 a	31.00 a	36.22 ab
Full SM + Bio	53.00 abc	24.00 bc	24.33 c	33.78 abc
Bio	51.00 abc	21.00 c	25.00 bc	32.33 c
IO	57.67 ab	24.33 bc	18.33 d	33.44 bc
Cut means	52.57	26.05	25.50	34.71
LSD (5%)	8.35	4.97	5.96	2.79

Statistically significant differences were found among the different letters in the same column. LSD: Least Significant Differences, SM: sheep manure, Bio: biofertilizer, IO: inorganic matter.

**Table 4:** Plant height and branch number of savory plants grown under different fertilizer treatments.

Treatments	Plant Height				Branch Number			
	1st Cut	2nd Cut	3rd Cut	Mean	1st Cut	2nd Cut	3rd Cut	Mean
50% SM	40.93 ^ns^	32.13 a	17.23 ^ns^	30.10 ^ns^	23.77 ab	8.63 ab	7.94 ^ns^	13.45 ab
Unfertilized	39.17	30.00 ab	14.97	27.82	21.73 b	8.17 ab	6.48	12.13 b
Full SM	39.13	29.77 ab	19.80	29.39	23.60 ab	9.27 ab	7.47	13.44 ab
50% SM + Bio	38.60	27.97 b	16.20	26.80	23.03 ab	9.17 ab	7.40	13.20 ab
Full SM + Bio	38.50	29.80 ab	18.30	29.09	25.93 a	9.70 ab	8.77	14.80 a
Bio	36.60	28.87 ab	18.18	28.73	24.10 ab	10.30 a	8.40	14.27 ab
IO	36.23	29.60 ab	17.81	28.00	23.53 ab	7.40 b	8.88	13.27 ab
Cut means	38.45	29.73	17.50	28.60	23.67	8.95	7.90	13.51
LSD (5%)	7.84	4.04	5.12	4.79	3.80	2.51	3.08	2.28

Statistically significant differences were found among the different letters in the same column. ns: not significant, LSD: Least Significant Differences, SM: sheep manure, Bio: biofertilizer, IO: inorganic matter.

**Table 5:** Fresh and dry weights of savory plants grown under different treatment fertilizers.

Treatments	Fresh Herb Weight				Dry Herb Weight			
	1st Cut	2nd Cut	3rd Cut	Total	1st Cut	2nd Cut	3rd Cut	Total
50% SM	10.19 ^ns^	3.83 a	2.32 ^ns^	16.34 ^ns^	2.01 ^ns^	0.64 a	0.53 ^ns^	3.18 a
50% SM + Bio	6.82	2.84 ab	1.49	11.15	1.28	0.53 ab	0.32	2.14 ab
Bio	7.10	2.11 b	1.56	10.77	1.31	0.36 b	0.35	2.02 ab
Unfertilized	7.23	2.38 ab	1.66	11.27	1.19	0.44 ab	0.36	1.99 b
Full SM	7.24	3.01 ab	2.30	12.55	1.58	0.51 ab	0.51	2.61 ab
Full SM + Bio	6.34	3.02 ab	1.55	10.91	1.17	0.51 ab	0.37	2.05 ab
IO	7.31	2.78 ab	2.16	12.25	1.40	0.46 ab	0.47	2.33 ab
Cut means	7.46	2.85	1.86	12.18	1.42	0.49	0.42	2.33
LSD (5%)	4.70	1.51	1.21	6.25	0.89	0.21	0.27	1.19

Statistically significant differences were found among the different letters in the same column. ns: not significant, LSD: Least Significant Differences, SM: sheep manure, Bio: biofertilizer, IO: inorganic matter.

**Table 6:** Essential oil contents (% *v*/*w*) of savory plants grown under different fertilizer treatments.

Treatments	1st Cut	2nd Cut	3rd Cut	Mean
%50 SM	1.61 ^ns^	1.80 ^ns^	0.26 c	1.22 ^ns^
50% SM + Bio	1.07	1.60	1.20 a	1.29
Bio	1.44	1.70	0.73 b	1.29
Unfertilized	1.12	1.49	0.52 bc	1.04
Full SM	1.33	1.71	0.62 bc	1.22
Full SM + Bio	1.69	1.62	0.79 ab	1.37
IO	1.78	2.01	0.51 bc	1.43
Cut means (%)	1.43	1.70	0.66	1.27
LSD (%5)	0.78	0.71	0.43	0.43

Statistically significant differences were found among the different letters in the same column. ns: not significant, LSD: Least Significant Differences, SM: sheep manure, Bio: biofertilizer, IO: inorganic matter.

**Table 7:** Major essential oil components (carvacrol, thymol and γ-terpinene) of savory grown under different fertilizer treatments.

Treatments	Carvacrol				Thymol				γ-Terpinene			
	1st Cut	2nd Cut	3rd Cut	Mean	1st Cut	2nd Cut	3rd Cut	Mean	1st Cut	2nd Cut	3rd Cut	Mean
50% SM	12.60 d	2.51 f	7.56 g	7.56 g	0.17 e	8.60 b	8.60 b	4.37 b	7.24 g	5.79 e	6.52 g	6.52 f
50% SM + Bio	18.76 b	4.42 e	14.38 f	12.52 e	1.07 b	0.61 d	0.61 d	0.58 f	7.90 f	3.18 f	7.43 f	6.17 g
Bio	6.97 g	14.20 c	20.50 d	13.89 d	0.34 d	0.12 g	0.12 g	0.36 g	8.75 c	6.88 c	7.72 e	7.78 d
Unfertilized	15.26 c	5.05 d	42.54 a	20.95 a	0.80 c	1.34 c	1.34 c	0.77 d	8.26 d	6.87 c	8.06 c	7.73 e
Full SM	8.41 e	0.18 g	17.91 e	8.83 f	0.83 c	16.09 a	16.09 a	5.68 a	8.81 b	7.24 a	7.82 d	7.96 c
Full SM + Bio	19.51 a	18.17 a	22.98 c	20.22 b	1.18 a	0.43 e	0.43 e	0.89 c	8.16 e	6.97 b	15.38 b	10.17 b
IO	7.78 f	16.13 b	26.74 b	16.88 c	0.84 c	0.34 f	0.34 f	0.72 e	9.00 a	6.09 d	16.05 a	10.38 a
Cut means	12.76	8.67	21.80	14.41	0.75	3.93	1.05	1.91	8.30	6.15	9.85	8.10

Statistically significant differences were found among the different letters in the same column. SM: sheep manure, Bio: biofertilizer, IO: inorganic matter.

**Table 8:** Major essential oil components (α-terpinene, cymol, α-bisabolene and α-phellandrene) of savory grown under different fertilizer treatments.

Treatments	α-Terpinene				Cymol				α-Bisabolene				α-Phellandrene			
	1st Cut	2nd Cut	3rd Cut	Mean	1st Cut	2nd Cut	3rd Cut	Mean	1st Cut	2nd Cut	3rd Cut	Mean	1st Cut	2nd Cut	3rd Cut	Mean
50% SM	2.12 f	5.64 a	3.88 c	3.88 c	2.81 e	5.05 a	3.93 e	3.93 c	3.13 f	3.74 b	3.44 g	3.44 e	2.92 f	4.35 d	3.64 d	3.64 e
50% SM + Bio	3.02 d	5.45 d	5.55 b	4.67 b	3.61 c	3.18 f	4.16 d	3.65 e	4.22 a	3.43 d	3.61 f	3.75 d	3.59 e	9.76 a	5.97 a	6.44 a
Bio	2.01 g	5.46 d	3.46 e	3.64 e	3.32 d	4.9 b	4.71 b	4.31 b	4.01 b	1.03 e	4.42 d	3.15 f	7.18 a	3.76 e	4.08 c	5.01 b
Unfertilized	3.75 c	5.59 b	1.32 g	3.55 f	2.04 f	4.27 d	0.04 g	2.12 f	3.43 e	3.52 c	5.29 b	4.08 b	5.76 c	7.16 b	0.03 g	4.32 d
Full SM	2.50 e	5.24 e	3.48 d	3.74 d	3.33 d	3.94 e	4.52 c	3.93 c	3.95 c	0.79 f	4.17 e	2.97 g	6.46 b	3.27 f	4.34 b	4.69 c
Full SM + Bio	6.14 a	5.51 c	10.02 a	7.22 a	4.48 b	4.8 c	1.85 f	3.71 d	3.63 d	3.53 c	4.82 c	3.99 c	2.87 f	5.93 c	0.15 f	2.98 f
IO	5.71 b	2.32 f	2.4 f	3.48 g	5.06 a	3.16 f	9.49 a	5.90 a	4.17 a	4.58 a	7.09 a	5.28 a	4.03 d	1.38 g	0.17 e	1.86 g
Cut means	3.61	5.03	4.3	4.31	3.52	4.19	4.1	3.94	3.79	2.95	4.69	3.81	4.69	5.09	2.63	4.13

Statistically significant differences were found among the different letters in the same column. SM: sheep manure, Bio: biofertilizer, IO: inorganic matter.

**Table 9:** Total phenolic and flavonoid contents of savory based on the cut extracts.

Treatments	TPC				TFC			
	1st Cut	2nd Cut	3rd Cut	Mean	1st Cut	2nd Cut	3rd Cut	Mean
50% SM	41.07 c	40.96 d	98.55 ab	60.19 b	34.94 b	10.75 c	40.48 bc	28.72 cd
50% SM + Bio	35.88 d	34.43 e	101.00 a	57.10 c	25.68 c	18.44 b	45.97 a	30.03 bc
Bio	30.36 f	42.65 c	96.87 b	56.62 c	31.99 b	26.73 a	32.13 d	30.28 abc
Unfertilized	50.45 b	29.56 f	90.21 c	56.74 c	45.61 a	11.94 c	38.13 c	31.89 ab
Full SM	58.22 a	52.04 a	83.13 d	64.46 a	31.70 b	26.71 a	41.66 b	33.36 a
Full SM + Bio	33.82 e	49.35 b	73.47 e	52.21 d	25.00 c	20.85 b	34.16 d	26.67 d
IO	33.31 e	51.12 a	69.41 f	51.28 d	20.98 c	18.1 b	27.88 e	22.32 e
Cut means	40.44	42.87	87.52	56.95	30.84	19.07	37.20	29.04
LSD (5%)	1.70	0.99	3.89	1.65	5.82	3.89	3.19	3.08
CV (%)	2.36	1.30	2.50	1.63	10.60	11.47	4.82	5.95

Statistically significant differences were found among the different letters in the same column. LSD: Least Significant Differences, SM: sheep manure, Bio: biofertilizer, IO: inorganic matter.

**Table 10:** DPPH and FRAP values of savory based on the cut extracts.

Treatments	DPPH				FRAP			
	1st Cut	2nd Cut	3rd Cut	Mean	1st Cut	2nd Cut	3rd Cut	Mean
50% SM	35.37 ab	47.70 bc	18.13 c	33.74 cd	36.11 f	28.06 e	41.63 d	35.27 f
50% SM + Bio	40.94 a	60.86 a	12.29 c	38.03 b	55.91 d	27.28 f	51.37 a	44.85 b
Bio	38.18 a	42.67 c	17.46 c	32.77 d	57.80 c	31.57 d	42.80 c	44.06 c
Unfertilized	41.31 a	52.89 b	20.41 bc	38.20 b	63.67 b	25.13 g	33.79 g	40.87 e
Full SM	24.58 b	51.83 b	9.44 c	28.62 e	69.64 a	58.64 a	43.88 b	57.39 a
Full SM + Bio	43.01 a	53.85 ab	48.52 a	48.46 a	54.06 e	33.15 b	36.40 f	41.20 e
IO	25.44 b	54.93 ab	30.84 b	37.07 bc	57.50 cd	32.36 c	39.74 e	43.20 d
Cut means	35.55	52.11	22.44	36.70	56.38	33.74	41.37	43.83
LSD (5%)	12.53	7.26	12.35	4.15	1.70	0.44	0.48	0.64

Statistically significant differences were found among the different letters in the same column. LSD: Least Significant Differences, SM: sheep manure, Bio: biofertilizer, IO: inorganic matter.

## Data Availability

All data represented in this work is contained within the manuscript.

## Supplementary Materials

The following supporting information can be downloaded at: https://www.mdpi.com/article/10.3390/biology14081096/s1, Table S1: Degrees of freedom, F statistic and *p* values of the examined properties. Table S2: Minor essential oil compositions of the savory grown under different treatments-1. Table S3: Minor essential oil compositions of the savory grown under different treatments-2. Table S4: Minor essential oil compositions of the savory grown under different treatments-3. Table S5: Minor essential oil compositions of the savory grown under different treatments-4. Table S6: Minor essential oil compositions of the savory grown under different treatments-5.

## Abbreviations

The following abbreviations are used in this manuscript:

FRAP	Ferric-Reducing Antioxidant Power
DPPH	1,1-diphenyl-2-picrylhydrazyl
SM	Sheep Manure
NS	Not Significant
LSD	Least Significant Differences
PCA	Principal Coordinate Analysis
IO	Inorganic Fertilizer
DW	Dry weight
FW	Fresh weight
CFU	Colony forming units
IAA	Indole acetic acid
GA	Gibberellic acid
