# Supplementary material for: Co-Application of Sheep Manure and Azotobacter Biofertilizer Enhances Growth, Yield, Essential Oil Profile, and Antioxidant Activity in Summer Savory
Source: Biology (Basel). 2025 Aug 21;14(8):1096. doi: 10.3390/biology14081096 (PMC12383741; doi:10.3390/biology14081096)
Supplement: Supplementary file 1 [file biology-14-01096-s001.zip › biology-3778034-supplementary.pdf]

Table S1. Degrees of freedom, F statistic and p values of the examined properties.

Properties	Cuts	DF	F value	P value	Properties	Cuts	DF	F value	P value
%50 flowering day	1	6	2.578	0.0767	Carvacrol	1	6	74076.590	0.0000
	2	6	3.738	0.0248		2	6	99942.688	0.0000
	3	6	5.373	0.0066		3	6	7485651.020	0.0000
	Mean	6	3.160	0.0426		Mean	6	162939.747	0.0000
Plant height	1	6	0.399	0.8656	Thymol	1	6	362.897	0.0000
	2	6	0.945	0.0499		2	6	113664.887	0.0000
	3	6	0.886	0.5340		3	6	143183.049	0.0000
	Mean	6	0.505	0.7936		Mean	6	48932.230	0.0000
Branch number	1	6	1.037	0.0448	γ-terpinene	1	6	1012.910	0.0000
	2	6	1.420	0.0284		2	6	7632.924	0.0000
	3	6	0.738	0.6297		3	6	1002481.976	0.0000
	Mean	6	1.309	0.0325		Mean	6	27930.469	0.0000
Fresh herb weight	1	6	0.671	0.6756	α-terpinene	1	6	7713.795	0.0000
	2	6	1.247	0.0350		2	6	30584.471	0.0000
	3	6	0.928	0.5091		3	6	495043.610	0.0000
	Total	6	0.927	0.5093		Mean	6	22478.245	0.0000
Dry herb weight	1	6	1.066	0.4332	Cymol	1	6	2724.564	0.0000
	2	6	1.582	0.0235		2	6	11981.364	0.0000
	3	6	0.917	0.5153		3	6	524274.732	0.0000
	Total	6	1.265	0.0342		Mean	6	16504.436	0.0000
EOC	1	6	1.199	0.3703	α-bisabolene	1	6	446.064	0.0000
	2	6	0.522	0.7812		2	6	33083.774	0.0000
	3	6	4.331	0.0148		3	6	94465.390	0.0000
	Mean	6	0.805	0.5849		Mean	6	7047.634	0.0000
DPPH	1	6	3.504	0.0307	α-phellandrene	1	6	8332.128	0.0000
	2	6	5.909	0.0045		2	6	53298.857	0.0000
	3	6	11.104	0.0003		3	6	370358.561	0.0000

	Mean	6	21.314	0.0000		Mean	6	28617.994	0.0000
FRAP	1	6	355.832	0.0000	Hexanal	1	6	13706.346	0.0000
	2	6	6296.080	0.0000		2	6	5.355	0.0067
	3	6	1326.810	0.0000		3	6	472.463	0.0000
	Mean	6	1044.877	0.0000		Mean	6	3864.336	0.0000
Total phenolic content	1	6	346.541	0.0000	1R-α-Pinen	1	6	11371.679	0.0000
	2	6	714.832	0.0000		2	6	54247.765	0.0000
	3	6	98.568	0.0000		3	6	183825.732	0.0000
	Mean	6	70.379	0.0000		Mean	6	17806.086	0.0000
Total flavonoid content	1	6	18.465	0.0000	2(10) Pinene	1	6	450.038	0.0000
	2	6	25.224	0.0000		2	6	11685.732	0.0000
	3	6	35.860	0.0000		3	6	49207.829	0.0000
	Mean	6	13.411	0.0001		Mean	6	2953.891	0.0000
dI-Limonene	1	6	4.708	0.0109	d-Mycrene	1	6	4422.564	0.0000
	2	6	6480.605	0.0000		2	6	60966.655	0.0000
	3	6	15395.415	0.0000		3	6	251530.537	0.0000
	Mean	6	11.926	0.0002		Mean	6	11198.350	0.0000
Sabinene	1	6	1770.949	0.0000	2-α-Pinen	1	6	1799.038	0.0000
	2	6	24952.941	0.0000		2	6	9603.121	0.0000
	3	6	81115.317	0.0000		3	6	38027.124	0.0000
	Mean	6	2164.437	0.0000		Mean	6	14483.434	0.0000
trans-sabinene hydrate	1	6	215.103	0.0000	Phytol	1	6	455.795	0.0000
	2	6	98655.328	0.0000		2	6	2250.958	0.0000
	3	6	6321.366	0.0000		3	6	1702.244	0.0000
	Mean	6	5721.485	0.0000		Mean	6	646.236	0.0000
a-Terpinolene	1	6	33.987	0.0000	Isospathulenol	1	6	101.128	0.0000
	2	6	13758.958	0.0000		2	6	8534.622	0.0000
	3	6	14479.098	0.0000		3	6	22042.537	0.0000
	Mean	6	2058.920	0.0000		Mean	6	692.307	0.0000

Eugenol	1	6	235.487	0.0000	cis-sabinene hydrate	1	6	80.115	0.0000
	2	6	717.588	0.0000		2	6	14056.134	0.0000
	3	6	1225.098	0.0000		3	6	2177.268	0.0000
	Mean	6	253.150	0.0000		Mean	6	853.448	0.0000
Crvacryl acetate	1	6	3734.590	0.0000	4-Terpineol	1	6	929.423	0.0000
	2	6	16426.238	0.0000		2	6	48627.832	0.0000
	3	6	3939.000	0.0000		3	6	28472.780	0.0000
	Mean	6	2892.697	0.0000		Mean	6	2802.451	0.0000
beta-caryophyllen	1	6	2751.256	0.0000	Carvacrol methyl ether	1	6	220.718	0.0000
	2	6	86838.655	0.0000		2	6	3047.597	0.0000
	3	6	18438.220	0.0000		3	6	5625.366	0.0000
	Mean	6	8471.834	0.0000		Mean	6	422.574	0.0000
Alloaromadenderene	1	6	631.154	0.0000	ledene	1	6	677.064	0.0000
	2	6	7877.546	0.0000		2	6	6588.192	0.0000
	3	6	19342.244	0.0000		3	6	14771.122	0.0000
	Mean	6	1171.609	0.0000		Mean	6	2032.312	0.0000
Myristicin	1	6	3347.244	0.0000	cis-a-bissabolene	1	6	204.205	0.0000
	2	6	74017.427	0.0000		2	6	46144.034	0.0000
	3	6	7565.854	0.0000		3	6	13012.024	0.0000
	Mean	6	6232.812	0.0000		Mean	6	4145.705	0.0000
Ent-spathulenol	1	6	1958.359	0.0000	p-Menth-3en-lol	1	6	69055.556	0.0000
	2	6	15650.723	0.0000		2	6	302.080	0.0000
	3	6	8299.976	0.0000		3	6	11861.854	0.0000
	Mean	6	2366.523	0.0000		Mean	6	18558.231	0.0000
Apiol	1	6	3370.538	0.0000	Neophytadiene	1	6	920.026	0.0000
	2	6	3983.597	0.0000		2	6	318.144	0.0000
	3	6	19796.195	0.0000		3	6	3929.268	0.0000
	Mean	6	4337.424	0.0000		Mean	6	943.813	0.0000
	1	6	1125.385	0.0000		1	6	204805.500	0.0000

6,7-Dimethoxy-3-hydroxybenzopyr	2	6	15543.168	0.0000	Phthalic acid	2	6	8.320	0.0010
	3	6	20938.024	0.0000		3	6	3121.171	0.0000
	Mean	6	1363.973	0.0000		Mean	6	17922.339	0.0000

Table S2. Minor essential oil compositions of the savory grown under different treatments-1.

Treatments	Hexanal				1R-a-Pinen				2(10) Pinene				d-Mycrene				2-a-Pinen				dI-Limonene			
	1st cut	2nd cut	3rd cut	Mean	1st cut	2nd cut	3rd cut	Mean	1st cut	2nd cut	3rd cut	Mean	1st cut	2nd cut	3rd cut	Mean	1st cut	2nd cut	3rd cut	Mean	1st cut	2nd cut	3rd cut	Mean
**50% SM**	0.02e	0.07bc	0.05e	0.05e	3.09c	2.71d	3.67b	3.15bc	1.21d	2.54c	2.18b	2.32a	1.51c	5.46a	3.86b	3.61a	1.91bc	0.27g	1.57c	1.25e	0.97bc	1.4d	1.62a	1.26cd
**50% SM + Bio**	0.06de	0.20a	0.23b	0.16b	2.6c	5.51a	3.8a	3.97a	1.56bcd	2.28d	1.34e	1.87bc	3.24b	3.1c	0.48e	2.27b	2.27ab	2.99d	2.84b	2.7bc	0.88c	2.10a	1.18c	1.53a
**Bio**	0.12bc	0.08bc	0.25a	0.15b	4.99ab	2.41e	2.69d	3.36abc	2.5a	2.79a	1.67c	1.29d	2.92b	1.12f	1.37d	1.8c	3.37a	1.99f	0.60d	1.99d	1.81a	1.12e	0.38e	1.31bcd
**Control**	0.08cd	0.06bc	0.17c	0.1cd	3.67bc	4.6b	0.03f	2.77c	2.42a	1.28e	0.17f	1.98b	2.85b	2.03e	0.01g	1.63c	2.69ab	6.15b	0.06f	2.97b	1.37abc	1.79b	1.42b	1.45abc
**Full SM**	0.17b	0.1bc	0.12d	0.13bc	5.43ab	2.05f	2.94c	3.47ab	2.24ab	1.22f	2.41a	1.96bc	3.6b	2.25d	5.38a	3.74a	2.98ab	6.49a	4.74a	4.74a	1.48ab	1.79b	1.19c	1.48ab
**Full SM + Bio**	0.1cd	0.11b	0.05e	0.09d	6.47a	3.95c	0.17e	3.53ab	1.48cd	2.64b	1.48d	1.73c	1.55c	3.17b	0.25f	1.66c	0.83c	2.48e	0.02g	1.11e	1.8a	1.63c	0.99d	1.24d
**IO**	6.06a	0.04c	0.05e	2.05a	0.29d	0.89g	0.16e	0.45d	2.15abc	0.55g	0.13g	0.94e	5.63a	0.15g	1.78c	2.52b	2.63ab	4.35c	0.23e	2.4c	1.83a	0.43f	0.30f	0.88e
**Cut means**	0.94	0.09	0.13	0.39	3.79	3.16	1.92	2.96	1.94	1.90	1.34	1.73	3.04	2.47	1.88	2.46	2.38	3.53	1.44	2.45	1.45	1.47	1.01	1.31
**LSD (5%)**	0.04	0.07	0.01	0.04	1.80	0.02	0.01	0.61	0.71	0.02	0.01	0.24	0.89	0.02	0.01	0.30	1.12	0.07	0.03	0.38	0.57	0.02	0.01	0.20

Statistically significant differences were found among the different letters in the same column. LSD: Least Significant Differences, SM: Sheep manure, Bio: Biofertilizer, IO: Inorganic matter

Table S3. Minor essential oil compositions of the savory grown under different treatments-2.

Treatments	Sabinene				trans-Sabinene Hydrate				a-Terpinolene				cis-Sabinene Hydrate				4-Terpineol				Carvacrol methyl ether			
	1st cut	2nd cut	3rd cut	Mean	1st cut	2nd cut	3rd cut	Mean	1st cut	2nd cut	3rd cut	Mean	1st cut	2nd cut	3rd cut	Mean	1st cut	2nd cut	3rd cut	Mean	1st cut	2nd cut	3rd cut	Mean
**50% SM**	1.58cd	3.34d	2.85b	2.59b	0.73a	1.16d	1.12d	1.00bc	0.36ab	2.62a	1.58a	1.52a	0.95a	3.03a	1.07a	0.76cd	1.55c	4.12d	3.22e	2.96de	1.25a	0.37g	1.12b	0.91d
**50% SM + Bio**	1.19d	3.71b	3.55a	2.82ab	0.27b	6.90a	1.16c	2.78a	0.26b	1.19c	0.64d	0.7d	0.62b	0.94d	0.89d	0.8bc	2.31bc	4.17c	3.3d	3.26cd	1.79a	1.06d	1.29a	1.38a
**Bio**	3.12ab	1.70f	2.78c	2.53b	1.07a	1.11e	1.23a	1.14b	0.58a	0.48f	0.65d	0.57e	0.50b	0.85f	0.81f	0.9b	3.49a	5.13b	4.11c	4.24a	1.38a	0.53f	1.28a	1.06bcd
**Control**	2.74b	3.24e	0.01g	2.00c	0.88a	1.57b	0.38g	0.94c	0.38ab	0.31g	0.28f	0.32f	0.68ab	1.06b	1.02b	0.68d	3.19ab	3.04f	2.91f	3.05de	1.35a	1.13c	0.51f	1cd
**Full SM**	2.47bc	3.77a	2.62d	2.95a	0.68ab	1.17d	1.18b	1.01bc	0.46ab	0.75d	1.42b	0.88c	0.62b	0.98c	0.91c	1.47a	3.49a	0.81g	4.37a	2.89e	1.62a	1.16b	1.09c	1.29ab
**Full SM + Bio**	3.73a	3.57c	1.66f	2.99a	0.68ab	1.36c	0.67f	0.90c	0.47ab	0.69e	0.47e	0.54e	0.53b	0.91e	0.85e	0.74cd	3.33a	3.98e	4.25b	3.85b	1.12a	0.70e	0.66e	0.83d
**IO**	3.21ab	1.04g	1.93e	2.06c	0.91a	0.92f	0.85e	0.89c	0.56a	1.63b	0.73c	0.97b	0.44b	0.66g	0.49g	0.9b	2.25c	5.29a	2.70g	3.41c	1.22a	1.41a	0.89d	1.17abc
**Cut means**	2.58	2.91	2.2	2.56	0.75	2.03	0.94	1.24	0.44	1.1	0.82	0.79	0.62	1.2	0.86	0.9	2.8	3.79	3.55	3.38	1.39	0.91	0.98	1.09
**LSD (5%)**	0.92	0.02	0.01	0.31	0.43	0.02	0.01	0.15	0.22	0.02	0.02	0.08	0.32	0.02	0.01	0.11	0.91	0.02	0.01	0.31	0.74	0.02	0.01	0.25

Statistically significant differences were found among the different letters in the same column. LSD: Least Significant Differences, SM: Sheep manure, Bio: Biofertilizer, IO: Inorganic matter

Table S4. Minor essential oil compositions of the savory grown under different treatments-3.

Treatments	Eguenol				Crvacryl acetate				beta-Caryophyllen				Alloaromadenderene				Myristicin				ledene			
	1st cut	2nd cut	3rd cut	Mean	1st cut	2nd cut	3rd cut	Mean	1st cut	2nd cut	3rd cut	Mean	1st cut	2nd cut	3rd cut	Mean	1st cut	2nd cut	3rd cut	Mean	1st cut	2nd cut	3rd cut	Mean
**50% SM**	0.34cd	0.21d	0.36b	0.3b	0.31ef	0.34d	0.40d	2.72c	0.75d	4.58b	2.85f	2.72c	1.04b	1.55c	1.55d	1.38b	1.16b	0.53d	1.13a	0.94d	0.44c	1.45c	1.05e	0.98c
**50% SM + Bio**	0.94a	0.24c	0.40a	0.53a	0.54d	0.69c	0.51c	3.19b	2.6b	3.65c	3.33d	3.19b	0.94b	1.14d	1.69c	1.26bc	1.52b	0.17f	0.25e	0.65e	0.75b	0.74e	1.45c	0.98c
**Bio**	0.15d	0.22cd	0.39a	0.25bcd	2.62b	0.93b	0.91a	3.34b	3.8a	2.2d	4.03a	3.34b	1.97a	0.87e	2.33a	1.72a	1.11b	5.39a	0.76b	2.42a	1.57a	1.72a	2.06a	1.78a
**Control**	0.62b	0.18e	0.03f	0.28bc	0.22f	0.68c	0.17g	2.02e	1.87c	1.64f	2.56g	2.02e	0.82b	1.12d	0.84g	0.93d	3.39a	0.13g	0.27d	1.26c	0.27c	0.59f	0.76g	0.54d
**Full SM**	0.53bc	0.86a	0.24c	0.54a	0.42de	3.05a	0.71b	2.51d	2.65b	0.97g	3.91b	2.51d	1.12b	0.11f	2.18b	1.14cd	0.97b	3.45b	0.24e	1.55b	0.77b	0.27g	1.89b	0.98c
**Full SM + Bio**	0.19d	0.29b	0.19d	0.22cd	2.13c	0.70c	0.35e	2.82c	2.76b	2.02e	3.68c	2.82c	1.04b	1.69b	1.02f	1.25bc	0.12c	0.24e	0.2f	0.19f	0.36c	1.41d	0.91f	0.89c
**IO**	0.15d	0.31b	0.13e	0.20d	2.93a	0.17e	0.32f	4.64a	4.00a	6.77a	3.16e	4.64a	1.97a	1.95a	1.27e	1.73a	0.15c	2.17c	0.55c	0.96d	1.45a	1.63b	1.28d	1.45b
**Cut means**	0.42	0.33	0.25	0.33	2.63	3.12	3.36	3.04	2.63	3.12	3.36	3.04	1.27	1.2	1.55	1.34	1.2	1.73	0.49	1.14	0.8	1.12	1.34	1.09
**LSD (5%)**	0.21	0.03	0.01	0.08	0.44	0.02	0.01	0.15	0.44	0.02	0.01	0.15	0.61	0.02	0.01	0.21	0.68	0.02	0.01	0.23	0.27	0.02	0.01	0.10

Statistically significant differences were found among the different letters in the same column. LSD: Least Significant Differences, SM: Sheep manure, Bio: Biofertilizer, IO: Inorganic matter

Table S5. Minor essential oil compositions of the savory grown under different treatments-4.

Treatments	cis-a-Bissabolene				Ent-Spathulenol				Apiol				6,7-Dimethoxy-3-hydroxybenzopyr				Isospathulenol				p-Menth-3en-lol			
	1st cut	2nd cut	3rd cut	Mean	1st cut	2nd cut	3rd cut	Mean	1st cut	2nd cut	3rd cut	Mean	1st cut	2nd cut	3rd cut	Mean	1st cut	2nd cut	3rd cut	Mean	1st cut	2nd cut	3rd cut	Mean
**50% SM**	0.69cd	2.62c	1.83b	1.71b	1.83c	0.8d	1.77a	1.46cd	2.05a	0.12e	1.59a	1.25ab	0.73d	2.19c	1.64b	1.52b	0.68ab	0.89c	0.95b	0.84b	1.65a	0.02c	1.24a	0.97a
**50% SM + Bio**	1.09bc	1.27e	1.7c	1.35d	4.41a	2.38b	1.13c	2.64a	1.57ab	0.89b	0.20c	0.89b	1.2c	2.58b	1.48c	1.75a	0.69ab	0.45f	0.43e	0.52c	0.77ab	0.01c	0.06de	0.28bc
**Bio**	1.55a	3.98b	1.37d	2.3a	2.92b	0.8d	1.31b	1.68c	0.36c	0.08f	0.08d	0.17c	1.69b	1.08f	0.85e	1.21c	0.83a	2.05a	0.32g	1.07a	0.01b	0.02c	0.05e	0.03c
**Control**	0.67d	1.12f	0.64g	0.81f	1.71c	2.6a	0.71e	1.67c	2.47a	1.12a	0.56b	1.38a	1.21c	1.82d	0.10g	1.04d	0.54ab	0.41g	2.01a	0.99a	0.64b	0.01c	0.4b	0.35b
**Full SM**	1.09bc	1.06g	1.27e	1.14e	2.73bc	2.36b	1.05d	2.05b	2.25a	0.37c	0.07de	0.9b	2.95a	1.69e	0.98d	1.87a	0.68ab	0.54e	0.38f	0.53c	0.89ab	0.05b	0.03f	0.32bc
**Full SM + Bio**	1.05bcd	1.73d	1.88a	1.55c	2.16bc	0.78d	0.69f	1.21d	0.47bc	0.02g	0.04f	0.18c	1.92b	0.51g	1.69a	1.37b	0.41b	0.85d	0.54c	0.6c	0.09b	0.02c	0.16c	0.09bc
**IO**	1.35ab	4.72a	0.98f	2.35a	3.20b	1.03c	1.14c	1.79bc	0.40bc	0.19d	0.06e	0.22c	1.85b	3.10a	0.65f	1.87a	0.64ab	1.65b	0.51d	0.93ab	0.03b	0.27a	0.07d	0.12bc
**Cut means**	1.07	2.36	1.38	1.60	2.71	1.54	1.11	1.79	1.37	0.40	0.37	0.71	1.65	1.85	1.06	1.52	0.64	0.98	0.73	0.78	0.58	0.06	0.29	0.31
**LSD (5%)**	0.41	0.02	0.01	0.14	1.07	0.02	0.01	0.36	1.20	0.02	0.01	0.41	0.43	0.02	0.01	0.15	0.40	0.02	0.01	0.14	0.95	0.02	0.01	0.32

Statistically significant differences were found among the different letters in the same column. LSD: Least Significant Differences, SM: Sheep manure, Bio: Biofertilizer, IO: Inorganic matter

Table S6. Minor essential oil compositions of the savory grown under different treatments-5.

Treatments	Neophytadiene				Phthalic acid				Phytol			
	1st cut	2nd cut	3rd cut	Mean	1st cut	2nd cut	3rd cut	Mean	1st cut	2nd cut	3rd cut	Mean
**50% SM**	1.16a	0.29d	1.01a	0.82a	0.75d	0.02d	0.57a	0.53b	0.81ab	0.20f	0.7a	0.57b
**50% SM + Bio**	1.06a	0.37c	0.38d	0.60ab	3.36a	0.02d	0.04f	0.65ab	1.11a	0.81b	0.33b	0.75a
**Bio**	0.27bc	0.21e	0.49c	0.32cd	0.06e	0.04bc	0.04f	0.18c	0.21c	0.28e	0.34b	0.28c
**Control**	1.04a	0.52a	0.65b	0.74ab	1.60c	0.05ab	0.17c	0.70a	0.85ab	1.07a	0.29c	0.74a
**Full SM**	0.85ab	0.36c	0.37d	0.53bc	2.92b	0.03cd	0.53b	0.65ab	0.82ab	0.61c	0.33b	0.59ab
**Full SM + Bio**	0.11c	0.20e	0.34e	0.22d	0.03e	0.06a	0.10e	0.18c	0.17c	0.28e	0.22d	0.22c
**IO**	0.24bc	0.49b	0.29f	0.34cd	0.05e	0.05ab	0.15d	0.34c	0.5bc	0.36d	0.20e	0.35c
**Cut means**	0.68	0.35	0.50	0.51	1.25	0.04	0.23	0.46	0.64	0.52	0.34	0.50
**LSD (5%)**	0.68	0.02	0.01	0.23	0.43	0.02	0.01	0.17	0.48	0.02	0.01	0.17

Statistically significant differences were found among the different letters in the same column.
LSD: Least Significant Differences, SM: Sheep manure, Bio: Biofertilizer, IO: Inorganic matte
